# Indicators of Visual Prognosis in Diabetic Macular Oedema

**DOI:** 10.3390/jpm11060449

**Published:** 2021-05-22

**Authors:** Sagnik Sen, Kim Ramasamy, Sobha Sivaprasad

**Affiliations:** 1 Department of Retina and Vitreous Services, Aravind Eye Hospital, Madurai 625020, India; sagnik@aravind.org (S.S.); kim@aravind.org (K.R.); 2 NIHR Moorfields Biomedical Research Centre, Moorfields Eye Hospital, 162, City Road, London EC1V 2PD, UK

**Keywords:** diabetic retinopathy, diabetic macular oedema, visual prognosis, indicators, personalized medicine

## Abstract

Diabetic macular oedema (DMO) is an important cause of moderate vision loss in people with diabetes. Advances in imaging technology have shown that a significant proportion of patients with DMO respond sub-optimally to existing treatment options. Identifying associations and predictors of response before treatment is initiated may help in explaining visual prognosis to patients and aid the development of personalized treatment strategies. Imaging features, such as central subfoveal thickness, photoreceptor integrity, disorganization of retinal inner layers, choroidal changes, and macular perfusion, have been reported to be prognostic factors of visual acuity (VA) in DMO. In this review we evaluated each risk factor to understand their relative importance in visual prognostication of DMO eyes post-treatment. Although individually, some of these factors may not be significant predictors, in combination they may form phenotypes that can inform visual prognosis. Stratification based on these phenotypes needs to be developed to progress to personalized medicine for DMO.

## 1. Introduction

Diabetic macular oedema (DMO) is the most frequent cause of moderate vision loss in people with diabetes. In 2019, there were approximately 28 million people with DMO globally [1]. For at least 40 years, patients and ophthalmologists were satisfied with the outcomes of the Early Treatment Diabetic Retinopathy Study (ETDRS) study that showed that the risk of moderate visual loss can be reduced by about 50% in laser treated individuals [2]. Over the last decade, intravitreal anti-vascular endothelial growth factor agents (anti-VEGF) have replaced macular laser and intravitreal steroids as the main treatment option for visual impairment due to centre-involving DMO (CI-DMO) [3]. Expectations of patients and providers have increased with the availability of anti-VEGF agents as approximately 50% of patients with visual impairment treated with anti-VEGF improve by two lines of visual acuity (VA) on ETDRS visual acuity charts by two years, if treated optimally [3,4]. However, DMO is not always associated with visual impairment, and some may resolve spontaneously or with treatment, while others progress to irreversible visual impairment. In this review, we evaluate the potential risk factors that can be considered in future prognostic models on visual impairment due to DMO, so that management of DMO can be personalised based on risk of visual loss. We broadly classified the prognostic factors into subjective and objective and then further grouped the imaging features into definite and possible, based on current evidence. However, the predicted visual outcome based on baseline prognostic factors may also be influenced by the drug given and treatment frequency.

## 2. Subjective Factors

### Presenting VA

Presenting VA is a definite prognostic marker. However, there are some caveats. Firstly, ceiling effect in eyes with good VA means that change in VA is not a good indicator of visual outcome in this group and the outcome in these eyes needs to focus on prevention of visual loss. Secondly, eyes with new-onset CI-DMO do not always present with visual impairment. However, if left untreated, visual deterioration may occur in some of these eyes. In DRCR Protocol V trial, 18.6% of eyes with good vision at presentation had a reduction of VA by 10 ETDRS letters or more after an observation period of 2 years and needed injections [5]. The exact timepoint or reason for incident visual impairment in these cases and not others remain unclear. Control arms of the RISE and RIDE studies also showed that on average, patients with visual impairment due to untreated DMO can remain stable for a further two years [6]. However, this group did not attain similar VA gains when initiated on ranibizumab after two years compared to those who were initiated early, suggesting that irreversible structural changes do occur if CI-DMO is left untreated and that these are not visible on OCT or probably, these characteristic changes have been unidentifiable to date [7]. When we synthesize the results of RISE and RIDE and Protocol V together, we can conclude that eyes with CI-DMO that experience visual deterioration over the two years of observation may not gain full visual potential compared to those who were started on anti-VEGF early. Although significant emphasis is made on presenting VA, these observations reinforce that structural changes precede decreases in visual function. However, further research is required to identify the imaging phenotype of this high-risk group that might explain irreversible visual loss while being observed for deterioration of CI-DMO.

On the contrary, in DMO eyes with visual impairment of 6/12 or worse (Snellen), baseline VA is a predictor of response to anti-VEGF treatment. The RESTORE study showed that patients with poorer starting VA had greater gains in VA with ranibizumab than those with better presenting VA [8]. The post-hoc analysis of Protocol T also confirmed that the treatment effect varied according to the baseline VA, after adjusting for both VA and CST and the interactions with treatment [9]. However, in real-life studies with ranibizumab, eyes with baseline VA less than 37 letters fail to achieve good VA that met driving standards. This outcome was also noted in a study on early response to aflibercept after 3 loading injections in real-life suggesting that there are more eyes with irreversible structural changes in eyes with VA less than 37 letters [10]. Based on available evidence, early treatment with anti-VEGF agents is recommended. Waiting for visual deterioration in eyes with CI-DMO with good presenting VA in routine clinical practice is itself a poor prognostic indicator of final visual outcome.

## 3. Objective Factors

### 3.1. Optical Coherence Tomography Imaging

#### 3.1.1. Central Subfoveal Thickness (CST)

A decrease in CST on OCT is the most reliable objective measure of anatomical treatment response of DMO. It is also the most common secondary anatomical outcome measure used to substantiate primary visual outcomes in clinical trials on interventions for DMO. However, reduction of CST does not mirror final VA outcome and is not a definite predictor of VA [11,12,13,14,15,16]. The RESTORE study shows that CST ≥ 400 µm resulted in more profound reduction of DMO with ranibizumab compared to laser, while the effects of macular laser and ranibizumab were similar in DMO eyes with CST < 400 µm [4]. However, the baseline predictors of the VIVID and VISTA study did not reveal such a cut-off. In fact, early treatment with aflibercept in eyes with CI-DME with CST < 400 µm resulted in significantly more gains in vision than those treated with laser [17]. Post-hoc analyses of RISE/RIDE and DRCR.net data suggest that continued treatment with frequent intravitreal anti-VEGF injections may improve functional outcome in non-responders who initially show suboptimal reduction of CST. These suggest that CST reduction has to be qualified with other morphological OCT features to predict visual outcome. For example, CST of similar thickness may have varying neuronal atrophy and Müller cell dysfunction that may relate better to visual outcomes [18]. Although CST is a prognostic indicator for fluid resolution in DMO, it is not a definite visual prognostic indicator.

#### 3.1.2. Morphological Phenotypes

There are several OCT morphological phenotypes of DMO. These include diffuse retinal thickening (DRT), cystoid macular oedema (CMO) and neurosensory detachment (NSD) [19]. These may be present in isolation or in combination. These features may affect different retinal layers. For example, sponge-like DRT may be seen with cystoid spaces present in the inner nuclear layer (INL) and outer plexiform layer (OPL) [20]. The cysts in CMO may be small to large in size and display variable fluorescein angiography findings ranging from honey-combing to petaloid pooling [20,21]. The size, location and angiographic appearance of cysts do not influence visual outcome in DMO.

However, association of cysts with other OCT features determine visual outcome. Cysts that are associated with photoreceptor damage negatively affect visual outcomes [22,23,24]. In addition, cysts that cause Müller cell dysfunction or loss are also detrimental to vision [25,26,27]. Early histological studies of DMO eyes have revealed that reversible edema develops from fluid accumulation within Müller cells [28]. The number of bridges between or within large cysts also determines VA. These bridges are presumed to be composed of Müller cells and bipolar cells [29]. Chronic fluid accumulation can lead to death of Müller cells and manifest as absence of bridging tissue between inner and outer retina. Therefore, although individually large cysts do not offer any prognostic significance, cysts in combination with ellipsoid zone loss or large cysts with lack of intervening bridges carry poor prognosis [30,31,32]. Large central cysts may also be a sign of macular ischaemia [33,34] but it is not a definite prognostic factor. Therefore, when we consider a prognostic model on cystoid spaces, the presence of large cysts with less bridging between inner and outer retina, foveal cysts, cysts with hyper-reflective material or hyper-reflective wall are all possible poor visual prognostic indicators [35,36,37].

Subretinal fluid (SRF) accumulation has been reported to be present in 15–30% of eyes with DMO [38]. The presence of NSD correlates with higher choroidal thickness, more hyperreflective foci, external limiting membrane (ELM) disruption but there are insufficient data to suggest that NSD is a visual prognostic marker.

#### 3.1.3. Photoreceptor Integrity

Photoreceptors may be affected by the overlying capillary non-perfusion or continual fluid accumulation can secondarily affect the outer layers [39,40]. Enhanced resolution of OCT has enabled distinct imaging of the photoreceptor inner segment/outer segment (IS/OS) junction, now known as the ellipsoid zone (EZ) [41]. The EZ marks the increased mitochondrial content in the photoreceptor inner segment ellipsoid, critical to photoreceptor function [41,42]. It is now understood that EZ discontinuity (loss of outer segments) precedes disruption of ELM (loss of photoreceptor cell bodies), EZ loss being representative of extensive photoreceptor cell body damage, with poorer visual prognosis [41,42]. The relative importance of ELM and EZ in preserving VA is unclear. An important consideration with the growing literature regarding the EZ is how to objectively and consistently evaluate disruption. Few studies have analyzed the percentage disruption of each layer, few have classified the EZ disruption into less than or more than 200 microns, while others have merely comment on the integrity [42,43,44,45]. Considering all the evidence on the effect of outer retinal changes on OCT on visual outcome, it can be concluded that a visible sub-foveal loss of ellipsoid layer or ELM and the percentage disruption are definite poor visual prognostic indicators [46,47,48,49,50]. 

Microperimetry data have revealed that loss of EZ may lead to 3.28 dB reduction in retinal sensitivity [51]. Some authors have found correlation between EZ disruption and significant reduction of macular sensitivity in DMO eyes. Wang et al. have reported the macular integrity index (MI) as a useful benchmark of reflecting functional status in DMO [52]. MI denotes the percentage of threshold drop to measure local functional deterioration, and has been found to be significantly and independently correlated with EZ disruption. However, one should bear in mind that visualisation of EZ and ELM in eyes with gross oedema may be challenging. Hence, the presence of subfoveal intact EZ and ELM are definite good prognostic indicators but their absence in the presence of gross oedema may not be a poor prognostic indicator and it is prudent to wait for fluid resolution before commenting on the integrity of EZ and ELM in these eyes.

#### 3.1.4. Intraretinal Hyperreflective Foci/Dots

Dot-like hyperreflective lesions within retinal layers have been described in OCT of DMO eyes, known as hyperreflective foci or dots (HRF/ HRD). These HRF have been proposed as lipoprotein exudates which pass into the interstitium after a breach in the inner blood-retinal barrier, and may possibly be precursors of hard exudates [53,54]. Hyperreflective foci may also indicate damaged photoreceptors and retinal pigment epithelium hyperplasia or metaplasia in other retinal diseases [54,55]. HRF are seen to be present initially in the inner retinal layers, from where they migrate into the outer layers. Subretinal HRF may end up as subfoveal hard exudates after resolution of NSD [56]. HRF present inside retinal cysts or lining cyst borders are believed to correspond to an advanced morphologic pattern of DMO which may be refractory to treatments [57,58]. Overall, HRF are believed to represent markers of inflammation in the retina [59].

A reduction of HRF has been seen with anti-VEGF injections [37,60]. The association of HRF and VA is controversial [53,60,61,62,63,64]. Whilst some reports suggest that eyes with HRF should be treated with intravitreal steroids, others have found similar response to either anti-VEGF or steroid. HRF responsive to anti-VEGF agents may be seen predominantly in the inner retinal layers; it has been proposed that the outer retinal layer HRFs may actually be a different entity. Anti-VEGF responsive inner retinal HRFs have been proposed to be of microglial origin [21,65]. On the contrary, the outer retinal HRFs, which are probable precursors of hard exudates, may not regress with treatment [56,66,67,68].

In summary, HRFs seem to be a bridge between the subclinical breakdown of the inner blood–retina barrier and the clinical swelling of retinal layers, and may indicate microvascular damage. However, more research is required to understand its relevance as a visual prognostic indicator [69]. Moreover, different reports have defined HRF based on their reflectivity either similar to the retinal pigment epithelium or to the surrounding tissue, and there is a need for standardization of the same [61,70].

#### 3.1.5. Disorganization of Retinal Inner Layers

Disorganization of inner retinal layers (DRIL) is believed to be a secondary morphological change of the retina when the synaptic connections in the bipolar, horizontal and amacrine cells are affected, leading to loss of transmission between the inner retina and outer retina [71]. In DMO, both ischaemia and inflammation may lead to neuronal and glial degeneration that result in DRIL [72,73]. Visual outcome depends on the intactness and organization of retinal pathways, and the presence of DRIL may be an indirect measure of intactness of the local neural connections in the retina. Unlike other OCT based morphological parameters of the inner retina that do not correlate well with functional outcomes, DRIL is a better predictor of visual outcome [20]. 

DRIL has been proposed as a robust and surrogate biomarker of visual function in existing or resolved DMO [71,74]. DRIL may not be specific for DR or DMO, and may be a common response to retinal stress in presence of ischaemia. However, it may not always be associated with poor prognosis, highlighting that there may be further ultrastructural changes within DRIL that may more accurately define visual outcome. The presence of DRIL may also be associated with other OCT changes like EZ and ELM disruption, enlarged foveal avascular zone (FAZ) on OCTA. DRIL is associated with other functional changes including subnormal multifocal electroretinogram, impaired contrast sensitivity and visual field tests [75,76,77,78,79,80,81].

DRIL at the parafoveal area may be associated with a worse baseline VA in DMO eyes. Presence of DRIL at initiation of treatment, new-onset DRIL or increase in DRIL have all been reported as poor prognostic indicator of visual outcome in DMO eyes [71,82,83]. Recently, authors have noted that for each 100-μm increase in DRIL, there is a reduction in VA by approximately six letters, that is more than one line on the ETDRS chart [84]. The baseline volume of the intraretinal fluid in DMO may also show positive correlation with DRIL, along with poorer final VA after treatment with bevacizumab [85]. More importantly, DRIL may reverse with anti-VEGF treatment and the amount of change over initial few months may affect the final VA, independent of CST [71,79,82,85].

It is important that DRIL reversal be re-examined. However, these findings show that there may be a minimum area of DRIL required before visual function is affected. Reversal of DRIL is suggestive of decompression of the retina or realignment of neuronal and glial cells after transient disorganization in DMO. The definition or grading of DRIL in eyes with DMO may be challenging. Previous studies have assessed DRIL using SDOCT imaging via a Heidelberg Spectralis system (Heidelberg Engineering, Heidelberg, Germany), using a standard imaging protocol of 49 B-scans spanning a 20 × 20 frame in high-resolution mode [79]. Investigators have observed good correlations among DRIL and other OCT variables on different OCT platforms [86]. Understanding the variability in estimation of DRIL extent and ways to measure the reversal of DRIL may help us set meaningful thresholds in regard to its correlation with functional outcomes in DMO eyes. In summary, the presence of parafoveal DRIL is a definite poor visual prognostic indicator.

#### 3.1.6. Choroid

There are contradicting reports on the changes in choroidal thickness and DMO. Firstly, investigators have shown increased choroidal thickness in DMO in keeping with the fact that choroidal vasculature is dependent on VEGF [87]. A higher choroidal thickness at baseline and its response to anti-VEGF may be a surrogate for good response to treatment [88]. Conversely, a thick choroid post anti-VEGF may also indicate an eye that is a poor responder to anti-VEGF and an indicator of chronicity of DMO. However, thick choroid is not an indicator for DMO recurrence or higher number of injections [89]. On the contrary, other investigators have suggested that choroidal blood volume, flow and velocity decreases in diabetic eyes with DMO, which may lead to hypoxia of the outer retina, thereby leading to increase in VEGF levels [90,91,92]. The choroid in DMO is also not affected by systemic factors such as increased HbA1c, blood pressure, cholesterol or abnormal renal function [93]. At the present time, there is insufficient evidence to suggest that changes in central choroidal thickness, subfoveal choroidal thickness or choroidal vascularity index influence visual outcome in DMO [94,95,96,97,98,99,100,101,102,103,104].

### 3.2. OCT-Angiography

Optical coherence tomography angiography (OCTA) is a noninvasive technique by which signals detected from blood flow in retinal and choroidal vasculature can be used to yield blood flow maps and quantify retinal perfusion in all vascular layers of the retina and the choroid [105]. Currently, the delineation of the capillary plexuses is done automatically by each OCTA software into the superior capillary plexus (SCP) which is embedded in the ganglion cell layer and nerve fiber layer, the deep capillary plexus (DCP) in the inner nuclear layer, and the choriocapillaris (CC).

Macular oedema may affect the reproducibility and effectiveness of OCTA in the measurement of FAZ area, perimeter and circularity, flow in CC and vascular density (VD) indices, especially in DCP [106,107]. Non-perfusion areas in DCP with VD reduction and flow-voids in CC layer correspond to photoreceptor/ellipsoid zone disruption in macular ischemia cases [108,109,110]. If the resolution of DMO persists for over 12 months, photoreceptors may show long-term recovery along with improved visual outcome, especially in eyes which have better baseline DCP integrity [111]. Thus, baseline VD of DCP is a predictor of photoreceptor recovery (ellipsoid zone integrity) and subsequent visual outcome. Moreover, areas of ischemia or capillary dropouts on OCTA may correspond to areas of lower macular sensitivity on microperimetry [112]. DMO eyes may have more microaneurysms (MA) in both the capillary layers, with poor responders to anti-VEGF showing more MAs in DCP [113,114]. The significance of the location of MAs in SCP versus DCP is unclear. However, as DCP also contributes to the blood supply of the photoreceptor layer, MAs in the DCP may be associated with loss of outer retinal integrity compared to MAs in SCP, which is associated with poorer visual outcomes. However, further research is required in this area [115,116,117].

RISE/RIDE study data suggest that anti-VEGF is efficacious in DMO irrespective of macular perfusion status at baseline [118]. There is no difference in VD of SCP and DCP in both treatment responders and non-responders in DMO, and there is no correlation between treatment response and baseline or final VD, suggesting that macular perfusion may not be a predictor of treatment response [119]. A higher resolution of DCP is required to evaluate if changes in DCP may be used as a prognostic marker for anti-VEGF responsiveness in DMO eyes [111].

The role of macular perfusion in final visual prognosis is certain but the best parameter to measure macular perfusion on OCT-A remains to be understood.

### 3.3. Colour Fundus Photography

The association of baseline diabetic retinopathy severity score (DRSS) score with visual outcome of patients with CI-DME is also of interest. Patients who show a limited early response to treatment may have better baseline VA, thinner CMT and lesser severity of diabetic retinopathy [120]. The overall impact of DRSS score on visual outcome of CI-DME patients treated in the VIVID and VISTA study showed that the VA outcomes did not differ based on baseline DRSS score stratified into ETDRS severity levels ≤ 43, 47, and ≥53 [120,121].

## 4. Other Factors Affecting Visual Outcome

### Role of Systemic Risk Factors

There is significant evidence that systemic risk factors such as hyperglycaemia, hypertension and hyperlipidemia need to be optimally controlled before and after the onset of DMO and this needs to be emphasized to each patient. However, people with optimally controlled systemic risk factors do develop visual impairment due to DMO and some with poorly controlled risk factors do not develop DMO throughout their whole diabetes history. There is insufficient evidence that suboptimal control of any of these systemic risk factors influences visual prognosis due to DMO. These observations suggest that local ocular features are relatively more important in determining the visual status of eyes with DMO.

## 5. Conclusions

This review on factors determining visual impairment in DMO or visual prognostic factors in eyes post-treatment suggest the need for a systematic evaluation of both subjective and objective definite predictive factors in a well characterized cohort of sufficient sample size. The relative importance of these factors is unclear. Baseline VA is an important predictor of final visual gain, and patients need to be treated early, to prevent irreversible VA loss. However, even if patients present with visual loss at a later stage, initiating prompt treatment may still help preservation of remaining vision. OCT has significant importance in deciding initiation and continuation of treatment in DMO eyes. Although a change in central subfoveal thickness is a secondary outcome in clinical trials and is used for monitoring anatomical treatment response, it is not a visual prognostic indicator. Ultrastructural features, such as photoreceptor integrity and the presence of DRIL, may serve as better prognostic indicators. Although poor macular perfusion is likely to have a negative impact, further studies on it are required to ascertain the best parameter to measure macular perfusion. Systemic factors do not seem to have a significant role as a visual prognostic indicator. Although individual factors may not be of significance, the combination of these may form response phenotypes that are of prognostic significance. Prognostic models on visual impairment should be developed before personalised medicine can be provided for patients with DMO.

## Data Availability

Not applicable.
